# Investigating Fumarate Hydratase-Deficient Uterine Fibroids: A Case Series

**DOI:** 10.3390/jcm12175436

**Published:** 2023-08-22

**Authors:** Samar Alkhrait, Munira Ali, Elizabeth Kertowidjojo, Iris L Romero, Feighanne Hathaway, Obianuju Sandra Madueke-Laveaux

**Affiliations:** 1 Department of OBGYN, University of Chicago Medicine, Chicago, IL 60637, USA; iromero@bsd.uchicago.edu; 2 College of Medicine, University of Illinois, Chicago, IL 60607, USA; mmoham89@uic.edu; 3 Department of Pathology, University of Chicago Medicine, Chicago, IL 60637, USA; ekertowi@bsd.uchicago.edu; 4 Department of Medicine, University of Chicago Medicine, Chicago, IL 60637, USA; fhathaway@medicine.bsd.uchicago.edu

**Keywords:** fumarate hydratase deficiency, uterine fibroids, hereditary leiomyomatosis and renal cell cancer (HLRCC)

## Abstract

Uterine leiomyomas or uterine fibroids are the most common benign soft tissue tumor in reproductive-aged women. Fumarate hydratase deficient (FH-d) uterine fibroids are a rare subtype that is diagnosed only on pathologic evaluation. FH-d uterine fibroids may be the first indicator of hereditary leiomyomatosis and renal cell cancer (HLRCC) syndrome. Therefore, identifying and understanding the clinical implication and diagnosis of FH-d uterine fibroids is critical for early diagnosis of HLRCC. This case series investigates the uncommon yet significant condition of FH-d uterine fibroids. We examined the clinical manifestation, diagnostic imaging, and histopathological characteristics of FH-d uterine fibroids in five cases identified at our institution over the last ten years. All diagnoses were confirmed by pathologic evaluation after surgical treatment. Gynecologists and pathologists play a critical role in the early diagnosis of FH-d uterine fibroids and must recognize the relevant clinical and pathologic findings that raise suspicion about this diagnosis. The detection of these cases is largely dependent on the pathologist’s ability to recognize unique histopathologic features. Once these characteristics are identified, it should prompt a referral to a gynecologist to consider conducting germline genetic testing. The management of FH-d uterine fibroids necessitates a multidisciplinary approach, including proper genetic screening and regular surveillance, especially for renal tumors.

## 1. Teaching Points

1. Awareness of Similar Presentation: *FH*-d uterine fibroids often manifest similar clinical symptoms and imaging features as typical uterine fibroids, which can make their identification challenging. Therefore, meticulous patient history-taking becomes paramount, particularly noting any personal or family history of potential malignancies. This level of detail can help raise suspicion for conditions like *FH*-d uterine fibroids and HLRCC syndrome. It emphasizes the need for gynecologists to maintain a high index of suspicion and collaborate closely with pathologists who can confirm the diagnosis through histopathological examination. This collective approach can ensure early recognition and improved management of these cases, potentially preventing serious complications associated with these conditions.

2. Early Diagnosis and Management: Recognizing the importance of the early diagnosis of *FH*-d uterine fibroids is crucial to improving patient outcomes and minimizing risk. Gynecologists should be vigilant for any symptoms, such as abnormal or heavy bleeding and pelvic pain, while radiologists must be thorough in identifying any anomalies during imaging. Pathologists play a critical role in diagnosing *FH*-d uterine fibroids by identifying unique histopathological features, such as atypical cell structures or abnormal growth patterns. Close collaboration between gynecologists and pathologists is essential for formulating a timely and appropriate management plan, which may include surgical intervention or medical management, depending on the individual patient’s circumstances.

3. Multidisciplinary Approach: Given the rarity and complexity of *FH*-d UFs, a multidisciplinary team approach is crucial to provide optimal care. This team may include gynecologists, radiologists, pathologists, and genetic specialists, to ensure comprehensive evaluation, treatment, and follow-up care for affected patients.

4. Advancements in Research: Continued research into the pathophysiology, molecular mechanisms, and genetic factors of *FH*-d UFs is essential for the development of new diagnostic tools, targeted therapies, and preventive measures. This will contribute to improved patient care and better overall outcomes for individuals suffering from this rare and significant condition.

## 2. Introduction

Uterine leiomyomas, or uterine fibroids (UFs), are extremely common. Moreover, 20% to 50% of women will develop uterine leiomyomas by 30 years of age, and more than 80% of females may have uterine leiomyomas by 50 years [1]. Fumarate hydratase-deficient (*FH*-d) UF is a rare subtype of UF characterized by its association with both somatic and germline *FH* gene mutations. *FH*-d UF is a unique subset of UFs with distinct clinical implications [2].

Fumarate hydratase (*FH*) is an important enzyme of the tricarboxylic acid cycle, also known as the Krebs cycle. This enzyme is responsible for the conversion of fumarate to malate, which plays a crucial role in cellular energy production [3]. Mutations in the *FH* gene can lead to fumarate accumulation, which is hypothesized to contribute to tumorigenesis [4]. Hereditary leiomyomatosis and renal cell cancer (HLRCC) syndrome, also known as Reed syndrome, is a rare autosomal dominant hereditary tumor syndrome associated with pathogenic germline mutations of the *FH* gene located at chromosome 1q42.3-q43 [5]. Patients with HLRCC are predisposed to the development of cutaneous and uterine leiomyomas and, more seriously, a unique type of aggressive renal cell carcinoma [6]. A recent study suggests a dermatological examination once every two years as part of the surveillance program to monitor for the transformation of cutaneous leiomyoma (CLM) to leiomyosarcoma as CLMs were found to be the first manifestation of HLRCC and the most common symptom that led to the diagnosis [7].

This case series explores the clinical presentation, imaging, and histopathological features of *FH*-d UFs based on five clinical cases identified at our institution over the past decade. The rarity of this UF subtype underscores the importance of this investigation, as the accumulated experiences and insights from these cases can contribute to a deeper understanding of the condition and its management.

## 3. Case Presentations

**Figure 1:**
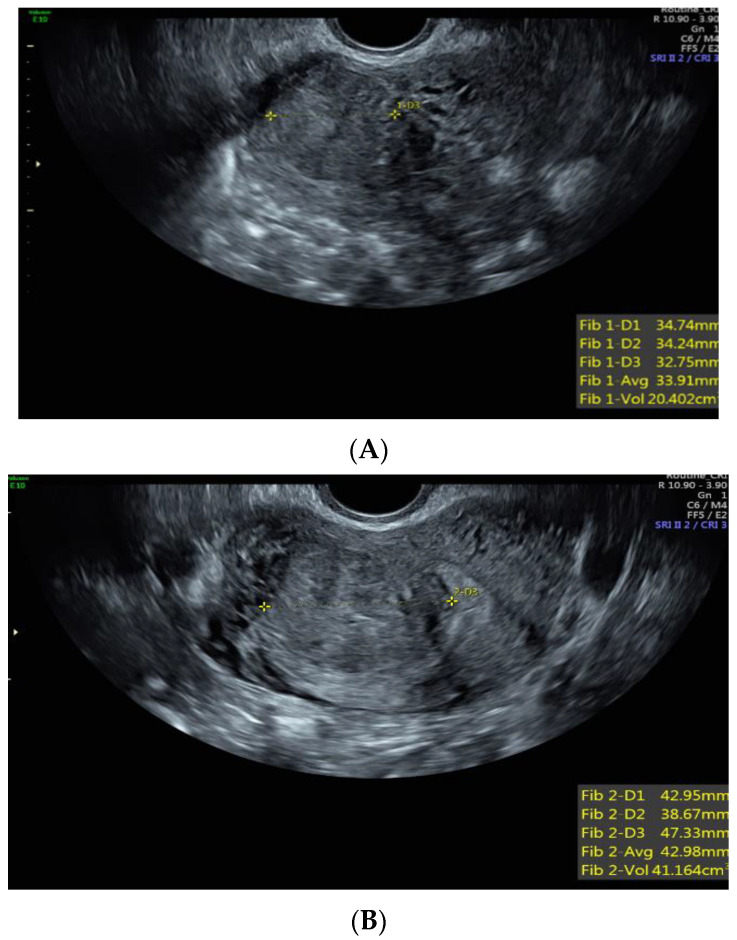
(**A**) Anterior intramural uterine fibroid. (**B**) Anterior submucous uterine fibroid.

Case 1: A 37-year-old White, non-Hispanic patient presented with heavy menstrual bleeding and pelvic pain. A pelvic ultrasound revealed a 4.5 cm intramural uterine fibroid (Figure 1A) and a 3.5 cm submucosal uterine fibroid (Figure 1B). She underwent a total laparoscopic hysterectomy with bilateral salpingectomy. Pathology confirmed a leiomyoma with features of *FH* deficiency; germline genetic testing was negative for known pathogenic mutations.

**Figure 2:**
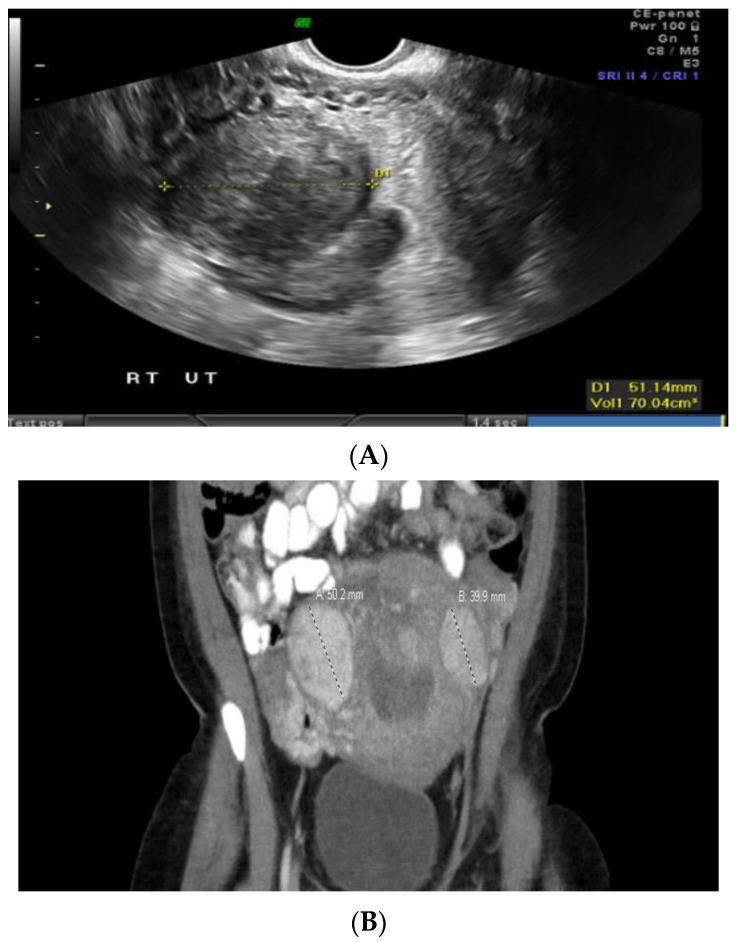
(**A**) Submucosal uterine fibroid. (**B**) Computed tomography (CT) scan-coronal section abdomen.

Case 2: A 34-year-old Black patient with a personal history of uterine fibroid and a family history of various cancers had an 8 cm submucosal uterine fibroid (Figure 2A). She underwent a total abdominal hysterectomy, left salpingo-oophorectomy, and right salpingectomy. Pathology revealed a leiomyoma with features of *FH* deficiency. Germline genetic testing showed a mutation in the *NTHL1* gene, which is not associated with leiomyomas or an increased risk for cancer, and a variant of uncertain significance in the *FH* gene, c.1055G>A (p.Gly352Asp). This specific missense change, guanine to adenine at nucleotide position 1055, results in an amino acid change from glycine to aspartic acid at codon 352. At the time, there was not enough evidence to know whether this change disrupts protein function; this specific missense variant had not been reported by other laboratories, nor had it been reported in the literature in individuals with *FH*-related conditions. However, given the family history of renal cancer, the patient was advised to undergo screening for this malignancy. A subsequent abdominal ultrasound and computed tomography (CT) scan revealed a large mass extending from the left kidney (Figure 2B). A needle core biopsy showed a high-grade renal cell carcinoma with papillary features, suggestive of *FH*-deficient renal cell carcinoma. The patient underwent radical left nephrectomy and adrenalectomy, which confirmed high-grade renal cell carcinoma with poorly differentiated areas and focal sarcomatoid spindle cell change, suspicious for *FH*-deficient renal cell carcinoma. Left retroperitoneal lymph nodes were diffusely involved by metastatic renal cell carcinoma.

Case 3: A 41-year-old White Hispanic patient presented with pelvic pressure and increased urinary frequency. An MRI of the pelvis showed a large uterine mass measuring 12.0 × 7.3 × 7.1 cm. She underwent a laparotomy and extensive myomectomy. After a few months, she returned with increased bladder pressure/pain, and an MRI of the pelvis revealed a 7.2 × 6.4 × 6.5 cm pelvic mass lesion, one of which potentially invaded the right external iliac vein. She underwent an exploratory laparotomy, total hysterectomy, and bilateral salpingectomy, which revealed a leiomyoma involving the right lower uterine segment and the right lateral wall of the cervix with exophytic extension into the right parametrial soft tissue with features of *FH* deficiency. Germline genetic testing was negative for known pathogenic mutations.

**Figure 3:**
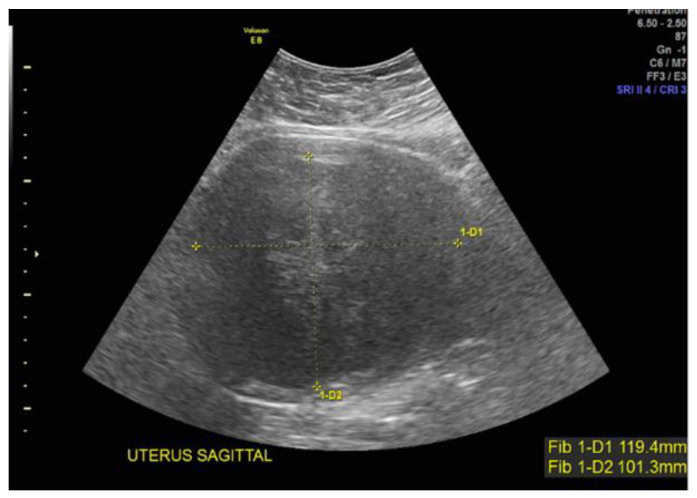
Subserous, fundal, homogeneous uterine fibroid.

Case 4: A 38-year-old Black patient presented with pelvic pain and heavy menstrual bleeding due to a 10 cm subserosal fundal uterine fibroid (Figure 3). She underwent a robotic hysterectomy with bilateral salpingo-oophorectomy, which confirmed a leiomyoma with features of *FH* deficiency; germline genetic testing was negative for known pathogenic mutations.

**Figure 4:**
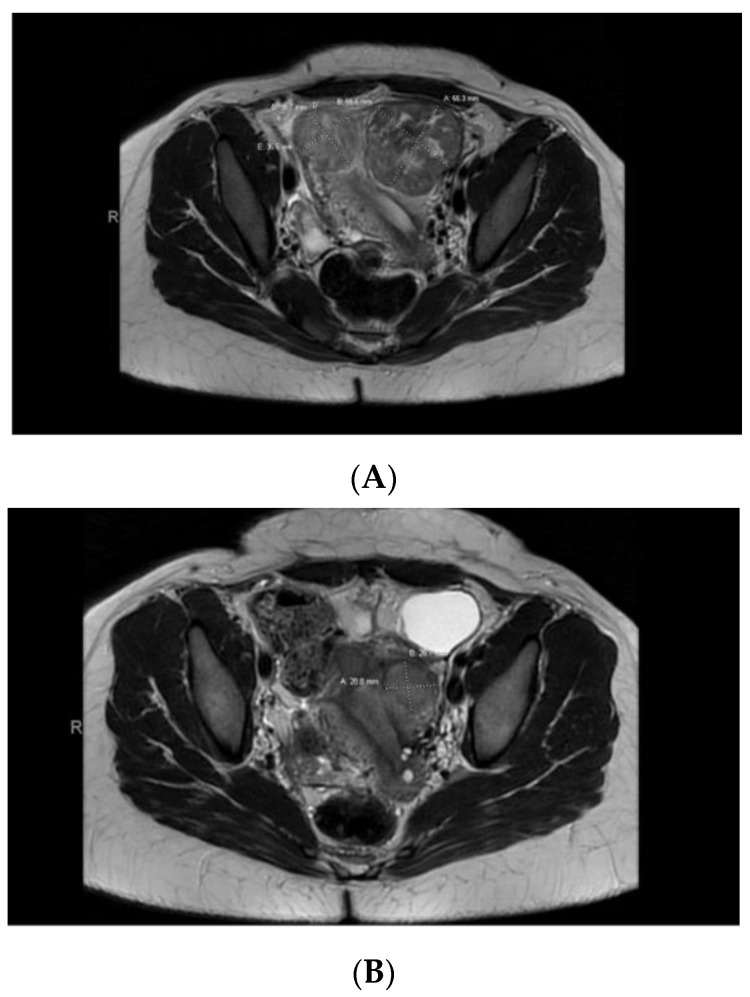
(**A**) Pelvic MRI pre-myomectomy. (**B**) Pelvic MRI post-myomectomy.

Case 5: A 32-year-old Black patient presented with increased pelvic pressure and urinary frequency. A pelvic ultrasound revealed a 7.5 cm UF and a subsequent pelvic MRI showed a dominant, primarily intramural, anteriorly situated UF measuring approximately 5.6 × 5.5 cm with central degeneration (Figure 4A). AdditionalUFs, including subserosal ones, were noted. The patient underwent a myomectomy with exploratory laparotomy, confirming a leiomyoma with *FH* deficiency features. Germline genetic testing was positive for a pathogenic mutation in the *FH* gene, c.239dupA (p.Ile81Aspfs*14). This mutation results in a premature stop codon in exon 2, causing disruption of protein production. A year later, the patient returned with pelvic pain, and a pelvic MRI showed her uterus contained at least 4 fibroids (with a dominant submucosal one), 2 in the right/fundus and 2 on the left (Figure 4B). She chose to undergo a robotic hysterectomy and bilateral salpingectomy, revealing additional *FH*-d leiomyomas.

## 4. Discussion

This case series presents five patients with diverse racial and ethnic backgrounds diagnosed with *FH*-d uterine fibroids. One patient had a germline pathogenic mutation in the *FH* gene, and one had a variant of uncertain significance (VUS) in the *FH* gene and subsequently developed renal cell carcinoma. Hereditary leiomyomatosis and renal cell cancer (HLRCC) is a disorder in which affected individuals tend to develop benign smooth muscle tumors (leiomyomas) in the skin and, in females, the uterus. This condition is also associated with an increased risk of renal cancer. In this disorder, growths on the skin (cutaneous leiomyomas) typically develop in the third decade of life. Most of these growths arise from the tiny muscles around the hair follicles that cause “goosebumps” [7]. They appear as bumps or nodules on the trunk, arms, legs, and occasionally on the face. Most women with HLRCC also develop uterine leiomyomas (fibroids). While UFs are common, women with HLRCC tend to have numerous large UFs that appear earlier than in the general population [8].

Approximately 10% to 16% of people with HLRCC develop a type of kidney cancer called renal cell carcinoma [7], with symptoms such as lower back pain, blood in the urine, or a palpable kidney mass. The average age of diagnosis for kidney cancer in HLRCC patients is in their forties [7,8]. These cases emphasize the importance of identifying *FH*-d uterine fibroids associated with HLRCC to appropriately manage affected patients.

It is essential to recognize that not all *FH*-d fibroids are the same. While sporadic *FH*-d fibroids are more common, syndromic *FH*-d fibroids are rare and often associated with hereditary leiomyomatosis [9]. Identifying characteristic histopathological features in young patients with moderate to large-sized, symptomatic uterine fibroids should lead to additional studies, including immunohistochemistry and genetic evaluation for germline pathogenic variants in the *FH* gene if needed. The pathologist should be expected to notice and correctly diagnose these histologic features, then perform stains and alert the gynecologist to pursue genetic testing.

In our series of five cases, no distinct or consistent ultrasound characteristics specific to *FH*-d UFs were identified. This observation aligns with the broader literature, where the ultrasound features of these rare fibroids have not been well-delineated and may not significantly differ from those of more common forms of UFs. This ambiguity in ultrasound imaging underscores the challenges in identifying *FH*-d UFs based on imaging alone and emphasizes the importance of maintaining a high index of suspicion for these lesions in patients presenting with certain clinical features or risk factors. Ultimately, the role of imaging in these cases may be more nuanced, potentially contributing to general fibroid detection and monitoring rather than acting as a definitive diagnostic tool for the identification of *FH*-d UFs.

In addition to the gynecological examination, all patients were thoroughly examined for skin changes, as cutaneous leiomyomas are a characteristic feature of HLRCC. Notably, no skin changes indicative of HLRCC were observed in any of the five patients.

Post-hysterectomy, all patients were closely monitored for potential renal malignancy, another association of HLRCC. The follow-up period ranged from 1–3 years post-surgery. It is significant to mention that one patient, who was diagnosed with HLRCC, was found to have renal malignancy one-year post-hysterectomy.

**Figure 5:**
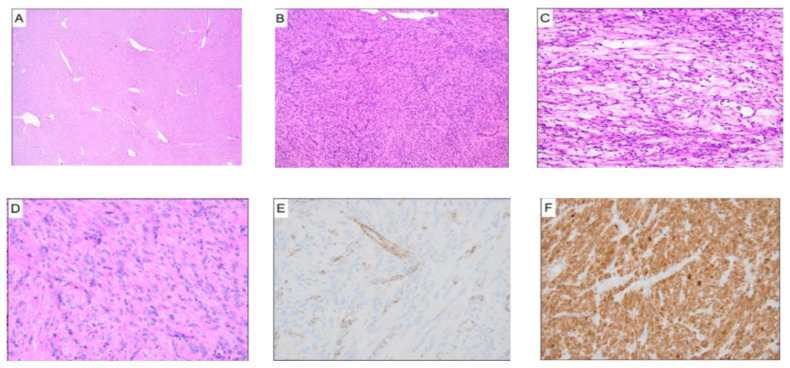
Pathology reports confirmed features of FH deficiency, characterized by the following histologic features: hemangiopericytoma-like or “staghorn” vasculature (**A**). linearly arranged nuclei (**B**) and areas of alveolar-type edema (**C**). At higher magnification, the nuclei are round-to-oval with prominent eosinophilic nucleoli, perinuclear clearing, and eosinophilic cytoplasmic globules (**D**). Immunohistochemistry shows a loss of FH staining with retained expression in endothelial cells (**E**) with positive staining for 2-S-succinocysteline (**F**).

In all five cases, pathology reports confirmed features of *FH* deficiency characterized by the following histologic features (Figure 5). Images were provided by the Pathology Department at the University of Chicago):

Previous studies have highlighted specific histopathological features that distinguish *FH*-deficient fibroids from other types of uterine fibroids [10]. Our observations reaffirm these findings, as our cases also demonstrated a common lack of bundling of tumor cells by collagenous bands and absent or poorly developed fascicular arrangements.

Gynecologists are crucial in the early diagnosis of HLRCC by identifying *FH*-d UF because timely diagnosis enables appropriate cancer surveillance and management for patients and their families. The presence of unusual skin lesions, early onset fibroid disease, numerous and large fibroids, extensive family history of early onset fibroids, and personal or family history of renal cell cancer should raise suspicion for HLRCC [11]. Emphasizing family history is advised if UFs show features suggestive of *FH* deficiency, supported by immunohistochemistry. Gynecologists should educate patients and encourage genetic counseling if this diagnosis is made.

By identifying germline *FH* variants, at-risk relatives can pursue testing. Proper screening and surveillance should begin once the diagnosis is established, especially for renal tumors [11].

Managing *FH*-d UFs requires a multidisciplinary approach and surveillance. Genetic counseling and testing are crucial, helping to identify germline mutations in the *FH* gene and enabling informed decision-making for patients and their families.

**Figure 6:**
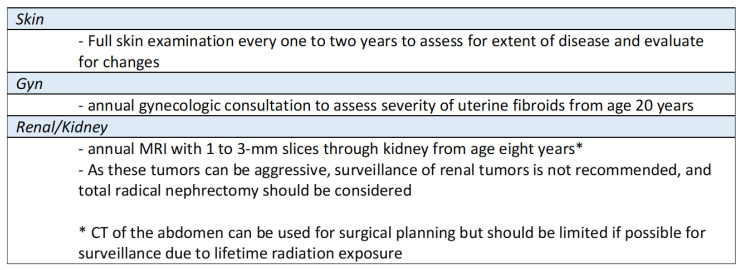
HLRCC·Management. Referenced with permission from the NCCN Clinical Practice Guidelines in Oncology (NCCN Guidelines^®^) for Guideline Name V.4.2023. © National Comprehensive Cancer Network, Inc. 2023. All rights reserved.

See a summary of HLRCC management in Figure 6 [12]:

## 5. Conclusions

In our investigation, we observed that *FH*-d UFs frequently mirror regular uterine fibroids in terms of clinical symptoms and imaging findings, which can make initial diagnosis challenging. This similarity underscores the vital role that thorough patient history-taking plays in the differential diagnosis process. However, in the case of *FH*-d UFs, a detailed medical and family history can provide crucial clues to the potential presence of this rare condition. Key indicators could include a history of early-onset or unusually severe uterine fibroids, the presence of cutaneous leiomyomas, or a family history of renal cell carcinoma, all of which could signal an underlying *FH* gene mutation.

This case series emphasizes the significant role gynecologists play in recognizing the distinctive features of *FH*-d UFs for early diagnosis of HLRCC and adequate treatment and surveillance. It recommends a holistic, interdisciplinary approach involving genetic counseling and testing to improve patient outcomes and inform decision-making regarding monitoring and treatment strategies. The case series also stresses the need for medical professionals to be more aware of the risks linked to *FH*-d UFs. It calls for further research to enhance our knowledge of the clinical implications, best management practices, and long-term outcomes for patients with *FH*-d uterine fibroids.

## Data Availability

No new data were created or analyzed in this study. Data sharing is not applicable to this article.
